# Ruptured mycotic aneurysm of intercostal arteries associated with vertebral osteomyelitis: a case report

**DOI:** 10.1186/s13019-023-02231-3

**Published:** 2023-04-17

**Authors:** Masatsugu Tsukamoto, Tadatsugu Morimoto, Tomohito Yoshihara, Masaaki Mawatari

**Affiliations:** Department of Orthopaedic Surgery, Faculty of Medicine, Saga University, Saga, Japan

**Keywords:** Intercostal artery pseudoaneurysm, Pyogenic spondylodiscitis, Mycotic aneurysm, Endovascular intervention, Massive hemothorax

## Abstract

**Background:**

Here, we report a rare case of massive hemothorax caused by rupture of an intercostal artery pseudoaneurysm associated with pyogenic spondylodiscitis, which was successfully treated with endovascular intervention.

**Case presentation:**

A 49-year-old man with schizophrenia, idiopathic esophageal rupture, postoperative mediastinal abscess, and pyothorax, diagnosed with pyogenic spondylodiscitis caused by methicillin-resistant *Staphylococcus aureus*. Magnetic resonance imaging and computed tomography (CT) showed extensive vertebral body destruction. The patient underwent a two-stage operation: anterior vertebral debridement and fixation with iliac bone graft and 10 days after first surgery, posterior fixation with instrumentation. Seven days after second surgery, the patient’s right chest pain increased, his blood pressure dropped, and he had shock. Chest X-ray showed massive hemothorax in the right lung. Chest CT and subsequent intercostal arteriography showed a pseudoaneurysm in the right T8 intercostal artery and active contrast extravasation from it. This seemed ruptured mycotic aneurysms involving intercostal vessels. These vessels were successfully embolized using micro-coils. Then, the patient completed the prescribed antimicrobial therapy in the hospital without any complications.

**Conclusions:**

Intercostal artery aneurysms are rare vascular abnormalities. They have the risk of rupture and may sometimes cause hemothorax and can be potentially life-threatening. Ruptured intercostal artery pseudoaneurysms are a good indication of endovascular intervention, and prompt embolization saved the life of the patient in this case report. This case report highlights the possibility of a ruptured intercostal mycotic aneurysm in patients with pyogenic spondylodiscitis and reminds physicians to be alert of this rare but potentially fatal complication.

## Background

Pyogenic spondylodiscitis (PS) is often associated with bacteremia and is commonly complicated by infective endocarditis (IE) [1]. Intercostal artery aneurysms are rare vascular abnormalities, usually secondary to prior trauma or surgery [2, 3]. Mycotic aneurysms in the intercostal region have been occasionally reported [4]. Here, we report a case of massive hemothorax caused by a ruptured intercostal artery pseudoaneurysm associated with PS, which was successfully treated with endovascular intervention. Informed consent was obtained from the patient. Clinical trial registration and institutional review board approval were not required and were waived for the purpose of this study.

## Case presentation

A 49-year-old man with schizophrenia was brought to our hospital as an emergency case of idiopathic esophageal rupture. The patient underwent an emergency laparoscopic surgery. No abnormal findings were found in the abdominal cavity. Dissection was proceeded from the left side of the esophagus and an abscess was observed. Perforation placement was searched, but the perforation site could not be found. Before the end of surgery, the mediastinum was irrigated with saline solution and the esophagus was covered with lesser omentum.

**Fig. 1:**
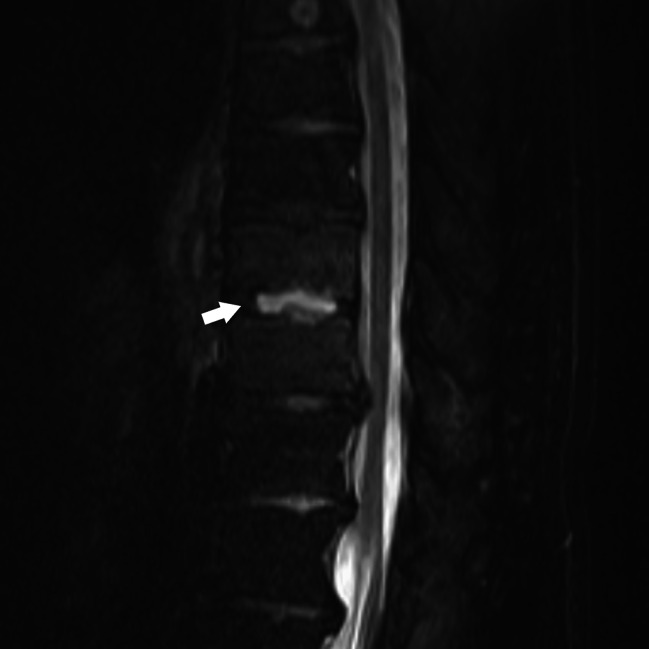
T2-weighted magnetic resonance imaging (MRI) image showing hyperintense signal in T9-10 disc space (white arrow)

However, after the surgery, the patient developed a mediastinal abscess and pyothorax, and two blood cultures were positive for methicillin-resistant *Staphylococcus aureus* (MRSA). Thoracic drainage and antibiotics improved the mediastinal abscess and pyothorax; however, the patient’s back pain and low-grade fever persisted. Magnetic resonance imaging (MRI) of the thoracic spine showed abnormal signal and extensive contrast in the vertebral bodies at T9 and T10, and high signal fluid retention on T2-weighteding imaging of the intervertebral disk at T9–T10 (Fig. 1). However, there were no findings of compression of the spinal cord. Based on the MRI findings, the patient was diagnosed with PS, and computed tomography (CT)-guided drainage was performed for disk drainage and detection of pathogenic bacteria. MRSA was detected in the bacterial culture of the intervertebral disk.

The patient was treated with intravenous daptomycin (700 mg/day) for 10 days, intravenous teicoplanin (800 mg/day) for 5 weeks, oral sulfamethoxazole–trimethoprim (4 g/day), and oral minocycline (200 mg/day).

The patient’s symptoms improved, and he was transferred to the hospital 3 weeks later. His oral antibiotic treatment was scheduled to continue; however, the patient stopped the medications at his own discretion.

**Fig. 2:**
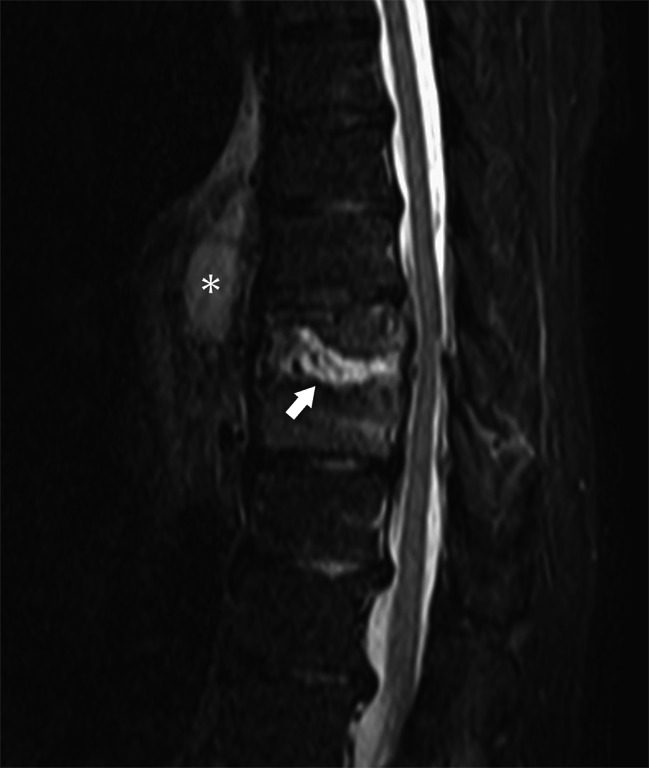
T2-weighted magnetic resonance imaging (MRI) image showing an enlarged hyperintense signal area in the T9/10 disc space (white arrow) and a hyperintense signal in the anterior part of the vertebral body that may be a paravertebral abscess(*)

After 6 weeks, the patient presented again with high fever and back pain. PS recurrence was suspected. MRI and CT showed an intradiscal abscess at the T9–T10 level, anterior paravertebral abscess, and extensive vertebral body destruction (Fig. 2). We diagnosed septic shock due to PS at the T9–T10 level.

The patient underwent a two-stage operation: anterior vertebral debridement and fixation with iliac bone graft and10 days after first surgery, posterior fixation with instrumentation.

**Fig. 3:**
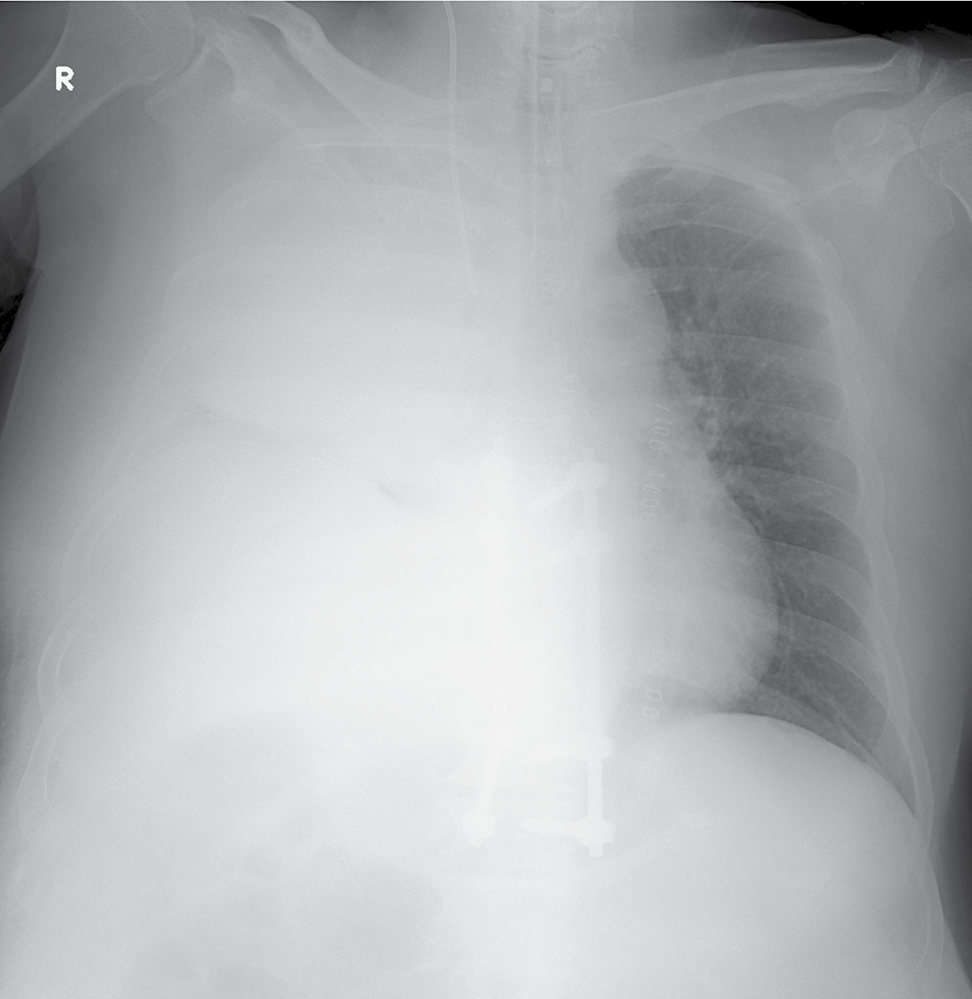
Chest plane radiograph shows massive hemothorax in the right lung

**Fig. 4:**
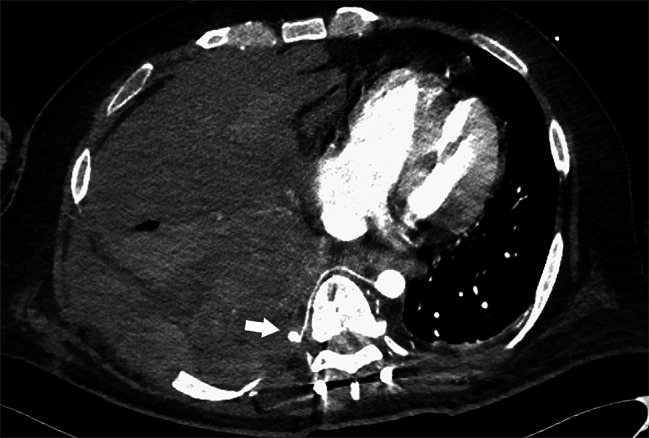
Contrast-enhanced computed tomography(CT) of the chest reveals a pseudoaneurysm in the right eighth intercostal artery (white arrow)

**Fig. 5:**
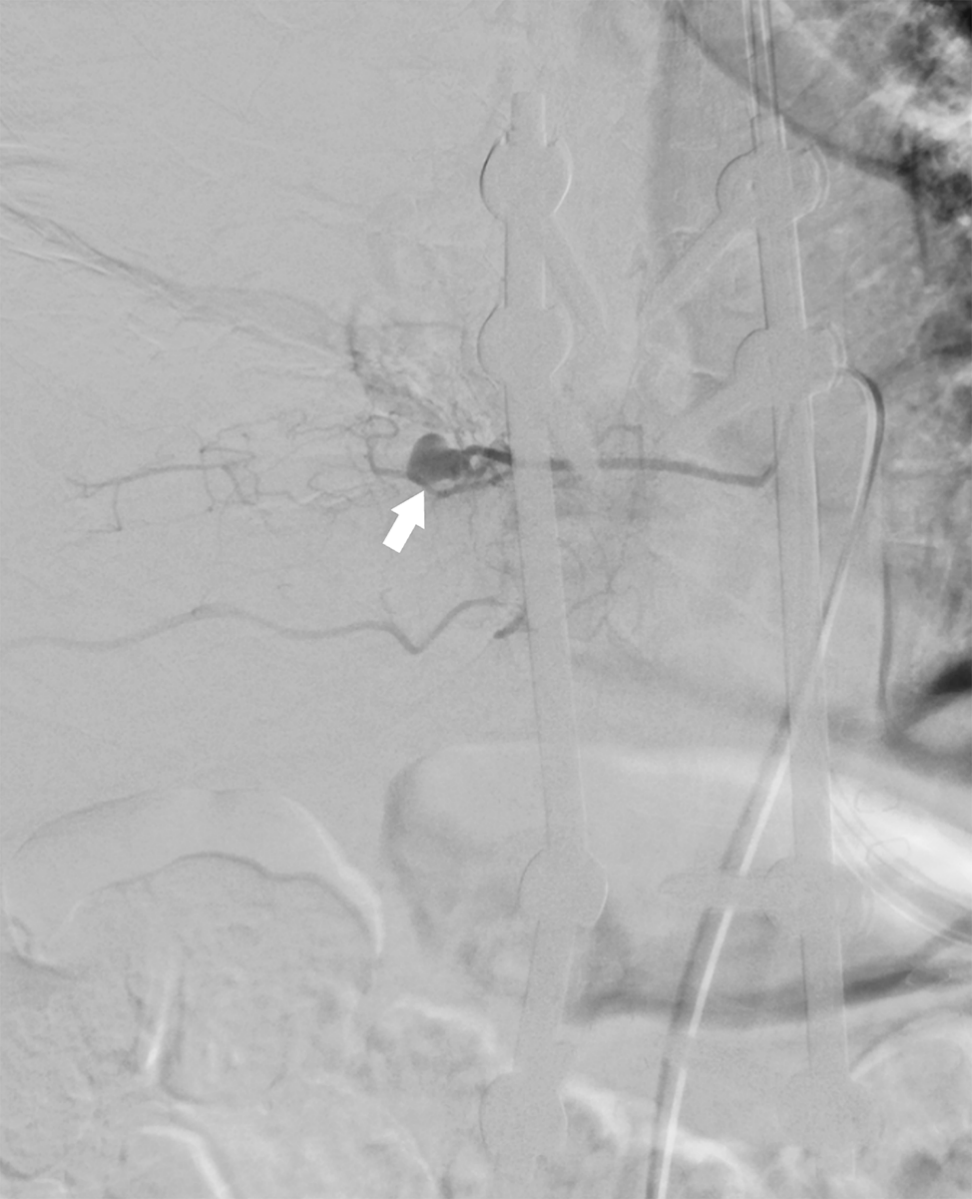
Intercostal arteriography revealed a pseudoaneurysm in the right eighth intercostal artery (white arrow)

**Fig. 6:**
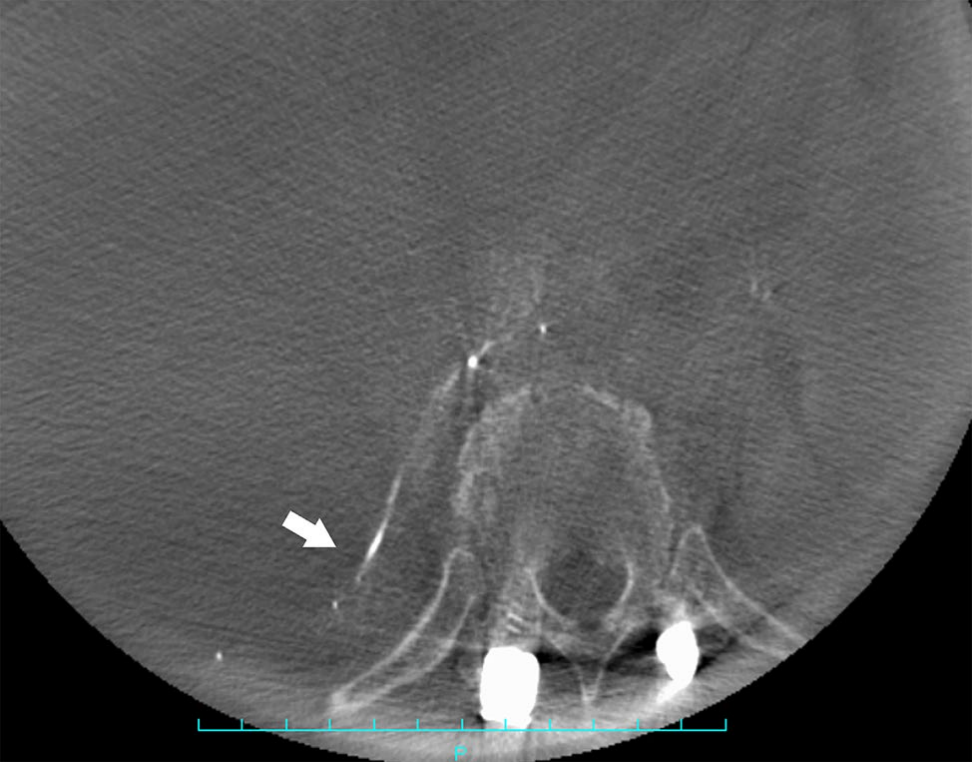
Computed tomography(CT) after N-butyl cyanoacrylate (NBCA) embolization showing that the eighth intercostal artery aneurysm was not apparent (white arrow)

Seven days after second surgery, the patient complained of increased right chest pain and shortness of breath. Chest X-ray showed a moderate pleural effusion in the right lung; however, electrocardiogram showed no obvious abnormality. Two days later, in the middle of the night, the patient’s right chest pain increased, his blood pressure dropped, and he had shock. Chest X-ray showed massive hemothorax in the right lung (Fig. 3). The patient’s hemoglobin had dropped sharply from 8.1 g/L to 6.4 g/L. Chest CT (Fig. 4) and subsequent intercostal arteriography (Fig. 5A) showed a pseudoaneurysm in the right eighth intercostal artery, which was considered to be the source of the bleeding. This presentation appeared to be ruptured mycotic aneurysms involving intercostal vessels. Following local anesthesia with 1% Lidocaine, the right common femoral artery was punctured utilizing a single-wall technique. A 4-Fr sheath was introduced, and 4-French shepherd’s hook catheter (Terumo Clinical Supply, Tokyo, Japan) advanced selectively into the right 8th intercostal arteries. Angiography confirmed the target aneurysms of the 8th intercostal artery. A 1.7-French microcatheter (Prograde-α; Terumo Clinical Supply) was inserted and the aneurysms were then embolized with a N-butyl cyanoacrylate (NBCA)/Lipiodol mixture (1:2) (Fig. 6). There were no intraoperative complications.

**Fig. 7:**
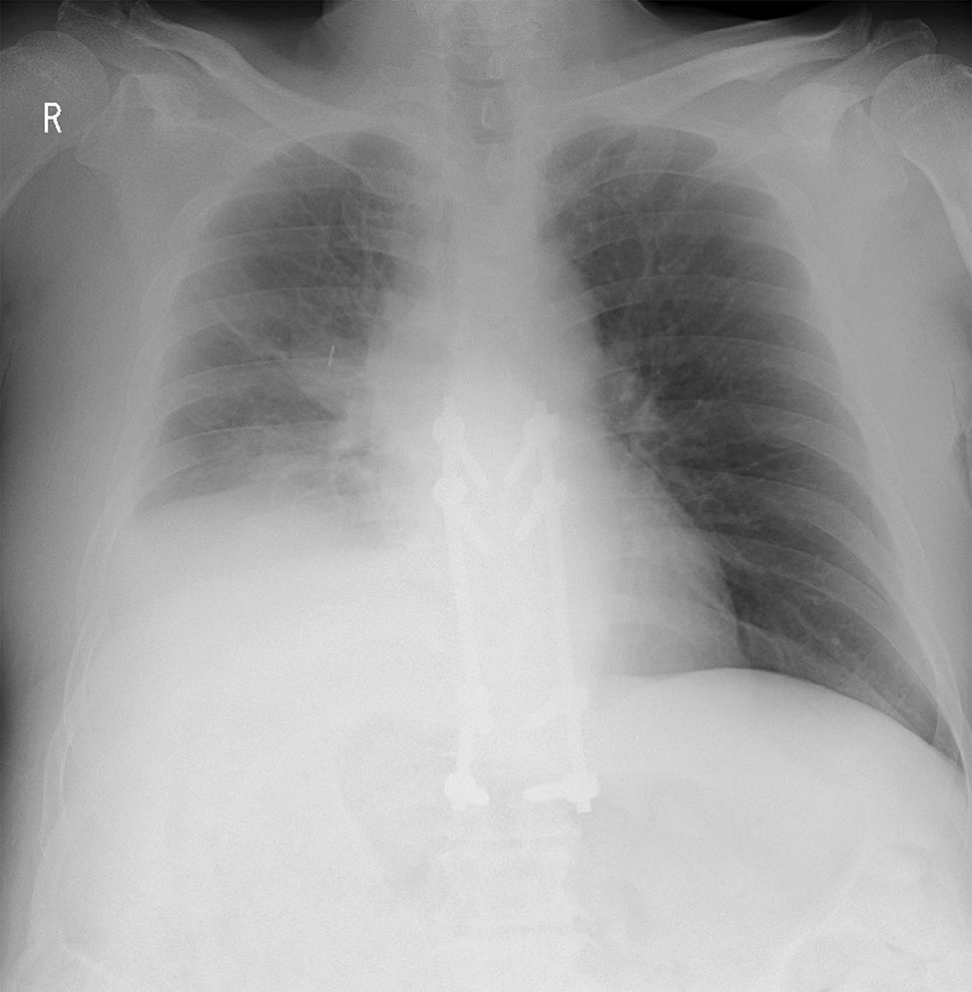
Chest plain radiograph shows the disappearance of a massive hemothorax in the right lung and improvement in lung field permeability

The patient was treated with intravenous daptomycin (700 mg/day) for 6 weeks, oral sulfamethoxazole–trimethoprim (4 g/day), and oral minocycline (200 mg/day). Then, the patient completed his course of antimicrobial therapy in the hospital without any complications (Fig. 7).

## Discussion and conclusions

Intercostal artery aneurysms are rare vascular abnormalities, which are typically diagnosed following rupture in patients with predisposing conditions, and have mostly been described through case reports [4–9]. These lesions have the risk of rupture and may sometimes cause hemothorax, as in the patient in this case report, and can be life-threatening.

Intercostal artery aneurysms are rare entities usually seen in connective tissue disorders, inflammatory conditions, and syndromes, such as Ehlers–Danlos syndrome, Kawasaki disease, and neurofibromatosis. Mycotic aneurysms in the intercostal region have occasionally been reported. Liu et al. [4] have reported a case of hemothorax due to ruptured mycotic aneurysms of intercostal arteries in a 40-year-old man with MRSA IE. Systemic septic emboli can be observed in 25–50% of patients with IE [10]. Conditions other than septic embolization from IE can cause mycotic aneurysms. Arterial injury can lead to direct inoculation of bacteria into the arterial wall, which can be observed in bacteremia, trauma, or catheter-based procedures or in intravenous drug users. Numerous cases of contiguous spread of local infection have also been reported, mostly in postoperative patients [11]. The etiology of mycotic aneurysms of intercostal arteries follows a similar pattern. In the patients in this case report, anterior fusion surgery was performed at the T9–T10 level; however, the T8 vertebra was not operated on. Therefore, the aneurysm was deemed caused by IE or direct spillover of infection, not surgical manipulation.

There are no established criteria for treating intercostal artery aneurysms. Fenwick et al. have reported successful endovascular management of a unique case of multiple idiopathic unruptured intercostal artery aneurysms. Appropriate diagnosis and prompt treatment of these rare vascular lesions are essential in preventing rupture. In contrast, ruptured intercostal artery pseudoaneurysms are a good indication of endovascular intervention. In this case report, the patient also had shock due to rupture; however, prompt embolization saved his life. Endovascular treatment should be the first choice even in ruptured cases.

Liu (2021) et al. [4] have reviewed 24 cases of intercostal artery aneurysms [6]. The symptoms included hemothorax in 10 patients, pulsatile mass in four patients, hemoptysis in two patients, hematoma in two patients, acute chest or back pain in three patients, and hematemesis in one patient. In this case report, the patient had chest pain a few days before shock caused by a ruptured aneurysm. If an intercostal aneurysm had been suspected and diagnosed at this point, rupture could have been prevented. Doppler ultrasonography and CT are the two most appropriate diagnostic modalities for evaluating intercostal artery pseudoaneurysms. Agarwal et al. anticipate these to be more commonly reported in the future, given the increased recognition and advent of multidetector CT imaging, and CT could depict an intercostal aneurysm in the patient in this case report [12]. Appropriate diagnosis and prompt treatment of these rare vascular lesions are essential in preventing rupture.

This case report highlights the possibility of ruptured intercostal mycotic aneurysm in patients with PS and reminds physicians to be alert of this rare but potentially fatal complication.

## Data Availability

The datasets used and/or analyzed during this study are available from the corresponding author on reasonable request.

## Abbreviations

IE: Infective endocarditis
MRSA: Methicillin-resistant *Staphylococcus aureus*.
MRI: Magnetic resonance imaging
CT: Computed tomography
PS: Pyogenic spondylodiscitis.
